# White matter alterations in autism spectrum disorder and attention-deficit/hyperactivity disorder in relation to sensory profile

**DOI:** 10.1186/s13229-020-00379-6

**Published:** 2020-10-19

**Authors:** Haruhisa Ohta, Yuta Y. Aoki, Takashi Itahashi, Chieko Kanai, Junya Fujino, Motoaki Nakamura, Nobumasa Kato, Ryu-ichiro Hashimoto

**Affiliations:** 1 Medical Institute of Developmental Disabilities Research, Showa University, 6-11-11, Kita-Karasuyama, Setagaya-ku, Tokyo 157-8577 Japan; 2 Department of Language Sciences, Graduate School of Humanities, Tokyo Metropolitan University, Hachioji, Japan

**Keywords:** Attention-deficit/hyperactivity disorder, Autism spectrum disorder, Developmental disorder, Diffusion tensor imaging, Sensory problem

## Abstract

**Background:**

Autism spectrum disorder (ASD) and attention-deficit/hyperactivity disorder (ADHD) have high rates of co-occurrence and share atypical behavioral characteristics, including sensory symptoms. The present diffusion tensor imaging (DTI) study was conducted to examine whether and how white matter alterations are observed in adult populations with developmental disorders (DD) and to determine how brain–sensory relationships are either shared between or distinct to ASD and ADHD.

**Methods:**

We collected DTI data from adult population with DD (a primary diagnosis of ASD: *n* = 105, ADHD: *n* = 55) as well as age- and sex-matched typically developing (TD) participants (*n* = 58). Voxel-wise fractional anisotropy (FA), mean diffusivity, axial diffusivity, and radial diffusivity (RD) were analyzed using tract-based spatial statistics. The severities of sensory symptoms were assessed using the Adolescent/Adult Sensory Profile (AASP).

**Results:**

Categorical analyses identified voxel clusters showing significant effects of DD on FA and RD in the posterior portion of the corpus callosum and its extension in the right hemisphere. Furthermore, regression analyses using the AASP scores revealed that slopes in relationships of FA or RD with the degree of sensory symptoms were parallel between the two DDs in large parts of the affected corpus callosum regions. A small but significant cluster did exist showing difference in association between an AASP subscale score and RD across ASD and ADHD.

**Limitations:**

Wide age range of the participants may be oversimplified.

**Conclusions:**

These results indicate that white matter alteration and their relationships to sensory symptoms are largely shared between ASD and ADHD, with localized abnormalities showing significant between-diagnosis differences within DD.

## Background

Autism spectrum disorder (ASD) is a developmental disorder characterized by impairment of social interaction and repeated restricted behavior [1]. Attention-deficit/hyperactivity disorder (ADHD) is also a developmental disorder with symptoms including attention-related difficulties and hyperactivity [1]. Despite the differences in their core symptoms, more than 50% of people with ASD have clinical ADHD symptoms [2, 3], while 20–30% of people with ADHD present with clinically significant symptoms of ASD [4, 5]. Furthermore, family members of individuals with one disorder are at risk of developing not only the one but also the other syndromes [6–8]. Such overlaps in symptoms and familial cross-aggregation have raised questions regarding similarity and distinction between these developmental disorders.

Neuroimaging studies contrasting either ASD or ADHD against typically developing people (TD) have shown that both disorders are characterized by atypicality in functional as well as in structural connectivity (reviewed in [9–13]). Thus, some prior studies using diffusion tensor imaging (DTI) have enrolled the three groups (ASD, ADHD, and TD) and reported on similarities and distinctions in white matter between the disorders compared with TD [14–16]. Results of categorical analyses contrasting the three diagnostic groups vary, possibly because of heterogeneity of the developmental disorders [17, 18]. However, they consistently emphasize the corpus callosum [14–16]. Besides categorical analysis, dimensional analyses were also conducted in these studies in which all the participants were allocated to one group and the relationship between DTI parameters and ASD symptoms was examined. Brain-ASD symptom relationships were reported in dimensional analyses across the diagnostic groups, suggesting people with ASD and with ADHD shared these relationships.

Sensory symptoms include both hyper- and hyposensitivity to textures, smelling, touching, visual, or auditory input (reviewed in [19]). Practically, sensory symptoms are one of the diagnostic criteria of ASD, but not for ADHD in DSM-5 [1]. Indeed, earlier studies reported that sensory symptoms were evident in more than 90% of people with ASD [20], while not being seen in many cases of ADHD [21]. However, more recent studies observed atypical sensory profiles in both pediatric [22] and adult populations [23], indicating that individuals with ADHD might suffer from sensory symptoms, perhaps to a lesser extent than ASD. Given that sensation is an input of external stimuli beginning at birth, sensory symptoms could underlie development of impaired social interaction [24]. In fact, sensory symptoms may cascade into higher-order dysfunction in individuals with ASD [19, 25].

As mentioned above, prior studies have examined the brain–symptom relationship across different clinical diagnoses with the perspective of ASD symptoms [14–16]. Although some prior studies have investigated white matter correlates of sensory processing symptoms [26, 27], to the best of our knowledge, no study has examined the similarity or distinction of the sensory symptoms between individuals with ASD and with ADHD. Given that in individuals with ASD, sensory symptoms may underlie the development of ASD symptoms (reviewed in [19]), investigation of relationships between sensory symptoms and the brain across diagnostic groups would deepen our understanding of whether ASD symptoms observed in individuals with ASD and with ADHD may share the same roots.

The current study tested three hypotheses. First, assuming that different diagnostic groups have different DTI parameters, we examined the effect of a diagnosis of ASD and ADHD on DTI parameters in 218 adults with or without developmental disorders to capture consistencies or inconsistencies in the results of prior studies. Second, we performed dimensional analyses to examine similarities in the brain–sensory symptoms relationship across diagnostic groups with a hypothesis that the brain–sensory relationship is independent of the clinical diagnosis. Finally, we conducted interaction analyses to see distinctions in brain–sensory symptoms relationships between diagnostic groups with an assumption that brain–sensory relationship is influenced by the clinical diagnoses. We selected data with small levels of head motion because it impacts the results of DTI analysis [28]. We used the Adolescent/Adult Sensory Profile (AASP) to assess sensory symptoms [29].

## Methods

### Participants

We recruited 160 adults with a primary diagnosis of ASD (*n* = 105) or ADHD (*n* = 55) and 58 TD participants, matched for age and sex. Clinical participants were recruited from the authors’ outpatient clinic at the Medical Institute of Developmental Disabilities Researches at Showa University, while TD participants were recruited via advertisement or acquaintances of the authors. After a multidisciplinary team, consisting of psychiatrists and psychologists, assessed all participants, clinical diagnosis of ASD and ADHD was made based on DSM-IV-TR. Among the 105 individuals with ASD, we administered the Autism Diagnostic Observation Schedule (ADOS) [30, 31] to 83 individuals. All participants in the ASD group who underwent the ADOS satisfied the diagnostic criteria for ASD. Among the ASD group, 5 individuals had substantial traits of ADHD. On the other hand, to exclude the comorbidity of ASD from ADHD, the ADOS was carried out in 21 out of 55 subjects in the ADHD group. Only one of the participants with ADHD met the diagnostic criteria for ASD using ADOS. However, after careful chart reviewing and clinical evaluation, the participant did not meet the DSM-IV-TR diagnostic criteria of ASD. Thus, we did not exclude the participant from the final analysis. The diagnosis of ADHD has been confirmed by administering Conners' Adult ADHD Diagnostic Interview for DSM-IV (CAADID) in all of the 55 participants in ADHD group [32]. To further characterize participants, the Autistic Spectrum Quotient (AQ) was obtained from 192 participants (ASD: *n* = 101, ADHD: *n* = 33, TD: *n* = 58) [33]. Data for assessment of ADHD severity were obtained from 150 participants (ASD: *n* = 86, ADHD: *n* = 46, TD: *n* = 18) using Conner's Adult ADHD Rating Scales (CAARS) [34]. The intelligence quotient (IQ) scores of the clinical participants (ASD: *n* = 105, ADHD: *n* = 44) were evaluated using either the Wechsler Adult Intelligence Scale-Third Edition (WAIS-III) or WAIS-Revised (WAIS-R) [35, 36]. As a measure for the education level, we collected final academic background of each patient: middle school graduate (1 ASD, 2 ADHD), high school graduate (38 ASD, 23 ADHD), university graduate (66 ASD, 30 ADHD). No significant group difference was found in the education level (*χ*^2^ (2) = 2.10, *P* = 0.35). Absence of a psychiatric diagnosis in TD participants was confirmed using the Mini-International Neuropsychiatric Interview [37]. In the TD group, IQ scores were estimated using a Japanese version of the National Adult Reading Test (JART) [38]. Each participant’s handedness was assessed using the Edinburgh Handedness Inventory. Fifteen participants were taking antipsychotics (13 ASD, 2 ADHD), while thirty-four participants had been administered stimulant (4 ASD, 30 ADHD). Full description of the medication the participants were taking at the time of the scan was provided in Additional file 1: Table S1. Exclusion criteria for all the participants included any history of head trauma, serious medical or surgical illness, or substance abuse. All the participants were confirmed to have a full-scale IQ above 74. Sensory symptoms were evaluated using the subscale of AASP [29] (ASD: *n* = 62, ADHD: *n* = 44, TD: *n* = 38). The AASP is a self-reported questionnaire consisting of 60 items from the following sensory sections, taste/smell processing, movement processing, visual processing, touch processing, activity level, and auditory processing. Participants were asked to respond to each item on a five-point Likert scale from “almost never” to “almost always.” Each item belongs to one of four subscales: Low Registration (hyposensitivity), Sensation Seeking, Sensory Sensitivity (hypersensitivity), and Sensation Avoiding. One participant failed to complete all the items included for Sensation Avoiding. These subscales were contrasted in the three groups using *F* tests. To correct multiple comparisons, we adopted the Bonferroni method and set the threshold for significance at *P* < 0.0125 (= 0.05/4: the number of subscales). The Institutional Review Board of Showa University Karasuyama Hospital approved all of the procedures adopted in this study. Written informed consent was obtained from all the participants after fully explaining the purpose of this study. The authors assert that all procedures contributing to this work comply with the ethical standards of the relevant national and institutional committees on human experimentation and with the Helsinki Declaration of 1975, as revised in 2008.

### Data acquisition

All magnetic resonance imaging (MRI) data were obtained using a 3 T MR Scanner (MAGNETOM Verio; Siemens Medical Systems, Erlangen, Germany) with a 12-channel head coil. Diffusion-weighted images were acquired using a single-shot, spin-echo, echo planar imaging sequence. The acquisition parameters were as follows: repetition time = 13,700 ms, echo time = 79 ms, field of view = 200 × 200 mm, matrix size = 100 × 100; 75 contiguous axial slices of 2.0 mm thickness without gap, phase-encoding direction = anterior–posterior, 65 non-collinear motion-probing gradients, and *b* = 1000 s/mm^2^. The directions of gradients were optimized according to a previous study [39]. The acquisition of the images included ten images without diffusion weighting (*b0*) interspersed throughout the sequence.

### Preprocessing

Images were preprocessed with FSL version 5.0 (FMRIB Software Library, https://www.fmrib.ox.ac.uk). DTI data are potentially at risk for a wide variety of artifacts, including motion artifacts and eddy current. Therefore, automatic artifact correction was conducted for all images using DTIPrep [40]. Susceptibility-induced distortion was corrected in all acquired images using TOPUP implemented in FSL [41, 42]. After the DTIPrep and TOPUP pipelines were performed, all data were registered to the first *b* = 0 image with affine transformation for correcting distortions. We used FSL rmsdiff functions to calculate root-mean-square (RMS) deviation of absolute intervolume displacement with respect to the first image of each run [43]. Because the results of comparisons of DTI parameters are particularly sensitive to head motion [28], participants with a maximum RMS over 2.5 mm were excluded from this study.

### Tract-based spatial statistics (TBSS) preprocessing

The images were then skull-stripped and fractional anisotropy (FA), mean diffusivity (MD), axial diffusivity (AD), and radial diffusivity (RD) images were calculated using the DTIFIT function for all participants. Generated FA images were registered to the Montreal Neurological Institute (MNI) 152 standard space using nonlinear registration. Normalized FA images were averaged to create a mean FA image, which was then thinned to create a mean FA skeleton [44]. Voxel-wise analyses using general linear models were conducted in skeleton areas with an FA of at least 0.2. Other DTI parameters, including MD, RD, and AD, were projected onto the mean FA skeleton.

### DTI group analyses

We performed an *F* test to examine the main effect of diagnosis on DTI parameters using the FSL randomize tool with age, sex, and motion as nuisance covariates. The contrasts were tested with 5000 permutations. The statistical threshold was defined at *P* < 0.05, correcting for multiple comparisons by threshold-free cluster enhancement (TFCE). We focused on clusters with a minimum size of 10 voxels. Post hoc pairwise group comparisons were made for clusters with a significant main effect of diagnosis on DTI parameters.

### DTI dimensional analysis

We performed the dimensional analyses to examine the relationships between sensory symptoms and DTI parameters. In these analyses, we used a vector of AASP subscale scores of all the subjects as an effect of interest. Here, we performed the analysis only for FA and RD maps because a significant main effect of diagnosis was not identified in the MD or AD map (see “Results” section). The analyses were conducted on voxels of the binary mask image identified by the *F* tests of FA and RD. The nuisance covariates included age, sex, and motion. The analysis was performed independently for all the four AASP subscales. The statistical threshold for significance was defined at *P* < 0.05, the TFCE corrected, and the spatial extension threshold was set to *k* > 10 voxels.

### DTI interaction analysis

We examined the interaction of the slope in relationships between the sensory symptoms (Low Registration, Sensation Seeking, Sensory Sensitivity, and Sensation Avoiding) and DTI parameters of FA and RD. The variables of interest were the element-wise product of the vector of an AASP subscale and the vector representing diagnostic status: (1) ASD or non-ASD, (2) ADHD or non-ADHD, and (3) TD or non-TD. The three vectors representing the diagnostic status (1–3) were also included in the model. The other nuisance covariates included age, sex, and motion. The analyses were conducted on voxels of the binary mask image identified in the *F* test and repeated for all the four AASP subscales. We adopted a threshold for statistical significance at *P* < 0.05, corrected for multiple comparisons using TFCE with a minimal number of voxels larger than 10.

### Supplementary analyses with a narrow age range with only males

To increase biological homogeneity, we repeated the analyses at cluster level with a narrow age range (i.e., 20–40 years old), focusing on only males. By narrowing the age range, the number of participants was reduced to 161 (78 ASD, 36 ADHD, 47 TD).

## Results

### Demographic data

Table 1 shows the demographic and clinical data of participants in the ASD, ADHD, and TD groups. *F* tests showed no significant differences in age, sex, or full-scale IQ (FIQ) among the three groups (*P* > 0.1). The main effect of diagnosis was observed in all of the four subscales of the AASP. Post hoc tests of Low Registration, Sensory Sensitivity, and Sensation Avoiding showed the same pattern when compared with TD; individuals with ASD and those with ADHD showed significantly higher scores, with no significant difference between these two clinical groups (Table 1). On the other hand, Sensation Seeking exhibited a different pattern in which individuals with ASD showed significantly lower scores compared to both the TD group and individuals with ADHD, which turned out not to be significantly different from each other.

**Table 1:** Descriptive statistics

Variable	ASD (*n* = 105)	ADHD (*n* = 55)	TD (*n* = 58)	*F* or *χ*^2^ statistic	Post hoc test
Age, mean, SD, y	31.2 (7.1)	31.2 (8.8)	29.4 (6.7)	*F* (2,215) = 1.2, *η*^2^ = 0.011, *P* = 0.31	NA
Male/female no	92/13	42/13	49/9	*χ*(2) = 3.41, *η*^2^ = 0.125, *P* = 0.18	NA
Handedness	76.8 (55.2)	75.5 (56.1)	87.4 (39.9)	*F* (2,215) = 0.97, *η*^2^ = 0.009, *P* = 0.38	NA
Head motion	1.06 (0.10)	1.05 (0.10)	1.07 (0.10)	*F* (2,215) = 0.42, *η*^2^ = 0.004, *P* = 0.66	NA
*IQ*					
Full	106.8 (14.8)	106.3 (12.5)	107.7 (7.7)	*F* (2,204) = 0.15, *η*^2^ = 0.001, *P* = 0.86	NA
Verbal	110.8 (15.0)	108.7 (14.1)		*F* (1,147) = 0.59, *η*^2^ = 0.004, *P* = 0.45	NA
Performance	100.1 (16.3)	102.0 (13.8)		*F* (1,147) = 0.45, *η*^2^ = 0.003, *P* = 0.50	NA
AQ	34.3 (6.0)	30.3 (8.5)	16.2 (5.8)	*F* (2,189) = 148.3, *η*^2^ = 0.611, *P* < 0.001	ASD > ADHD > TD
*CAARS*					
Inattentive symptoms	64.1 (14.3)	74.2 (12.4)	49.0 (8.3)	*F* (2,152) = 26.0, *η*^2^ = 0.255, *P* < 0.001	ADHD > ASD > TD
Hyperactive impulsive symptoms	59.4 (14.8)	67.2 (15.5)	49.9 (9.6)	*F* (2,152) = 10.3, *η*^2^ = 0.119, *P* < 0.001	ADHD > ASD > TD
ADHD symptoms total	63.4 (14.8)	72.9 (12.6)	49.4 (8.7)	*F* (2,152) = 21.3, *η*^2^ = 0.219, *P* < 0.001	ADHD > ASD > TD
*AASP*					
Low registration	36.9 (9.0)	39.1 (9.2)	28.3 (6.4)	*F* (2,141) = 18.7, *η*^2^ = 0.210, *P* < 0.001	ADHD = ASD > TD
Sensation seeking	31.8 (6.3)	38.1 (7.2)	40.8 (7.6)	*F* (2,141) = 22.6, *η*^2^ = 0.243, *P* < 0.001	ADHD = TD > ASD
Sensory sensitivity	39.1 (10.7)	41.8 (9.6)	32.9 (7.4)	*F* (2,141) = 8.97, *η*^2^ = 0.113, *P* < 0.001	ADHD = ASD > TD
Sensation avoiding	39.7 (10.1)	41.3 (10.2)	32.4 (7.0)	*F* (2,140) = 10.3, *η*^2^ = 0.129, *P* < 0.001	ADHD = ASD > TD

### DTI categorical analyses

**Fig. 1:**
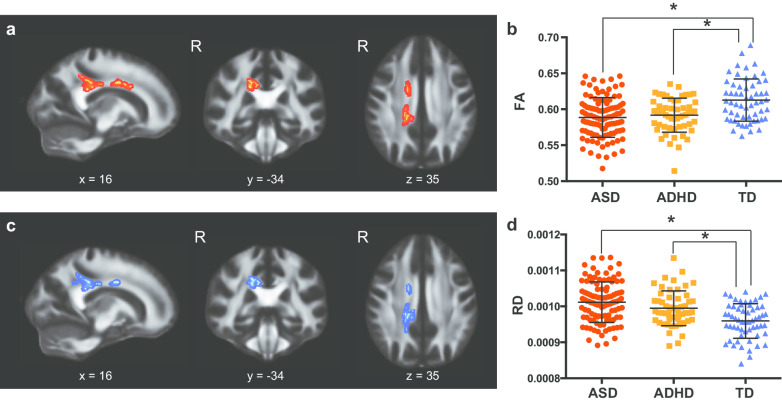
Significant main effect of diagnosis of developmental disorders on fractional anisotropy (FA) and radial diffusivity (RD). **a** Significant clusters of voxels showing a main effect of diagnosis on FA. **b** Plots of mean FA values extracted from significant voxels shown in **a**. We observed a significant main effect of diagnosis (*F* (2,217) = 15.54, *η*^2^ = 0.126, *P* < 0.001). **c** Significant clusters of voxels showing a main effect of diagnosis on RD. Note the high extent of spatial overlapping with **a**. **d** Plots of mean RD values extracted from significant voxels shown in **c**. We observed a significant main effect of diagnosis (*F*(2, 217) = 18.60, *η*^2^ = 0.147, *P* < 0.001). The asterisk indicates a significant difference between groups (*P* < 0.05)

The categorical analyses with FA showed the main effect of diagnosis on two clusters in the corpus callosum (Table 2 and Fig. 1a). Post hoc analyses revealed that individuals with ASD and with ADHD have significantly lower FA values compared with TD, whereas ASD and ADHD were comparable (Fig. 1b). The categorical analysis with RD also showed a main effect of diagnosis in the right posterior part of the corpus callosum (Table 2 and Fig. 1c). Post hoc tests using RD values extracted from the two clusters showed that, compared with TD participants, individuals with ASD and with ADHD had statistically significantly higher RD values, whereas there was no significant difference between ASD and ADHD (Fig. 1d). Other DTI parameters, such as MD and AD, did not show any significant main effect of diagnosis.

**Table 2:** Clusters showing significant effects of group

White matter tract		MNI coordinate			Cluster size
*F test*					
FA					
R. Posterior corpus callosum/corona radiata/cingulum		16	− 34	35	294
R. Body of corpus callosum		13	6	29	277
RD					
R. Posterior corpus callosum/corona radiata/cingulum		16	− 34	35	316
R. Body of corpus callosum		14	− 9	32	43
R. Body of corpus callosum		17	− 21	33	19
*Follow-up t test*					
FA					
TD > ASD					
R. Body of corpus callosum		11	6	27	277
R. Posterior corpus callosum/corona radiata/cingulum		16	− 36	27	264
TD > ADHD					
R. Body of corpus callosum		11	6	27	101
R. Posterior corpus callosum/corona radiata/cingulum		16	− 36	27	106
RD					
ASD > TD					
R. Posterior corpus callosum/corona radiata/cingulum		17	− 38	27	300
R. Body of corpus callosum		14	− 9	32	43
ADHD > TD					
R. Posterior corpus callosum/corona radiata		18	− 43	40	120
R. Body of corpus callosum		14	− 7	32	42
R. Cingulum		9	− 36	33	15
ASD > ADHD					
R. Posterior body of corpus callosum		17	− 33	34	61

### DTI dimensional analyses

**Fig. 2:**
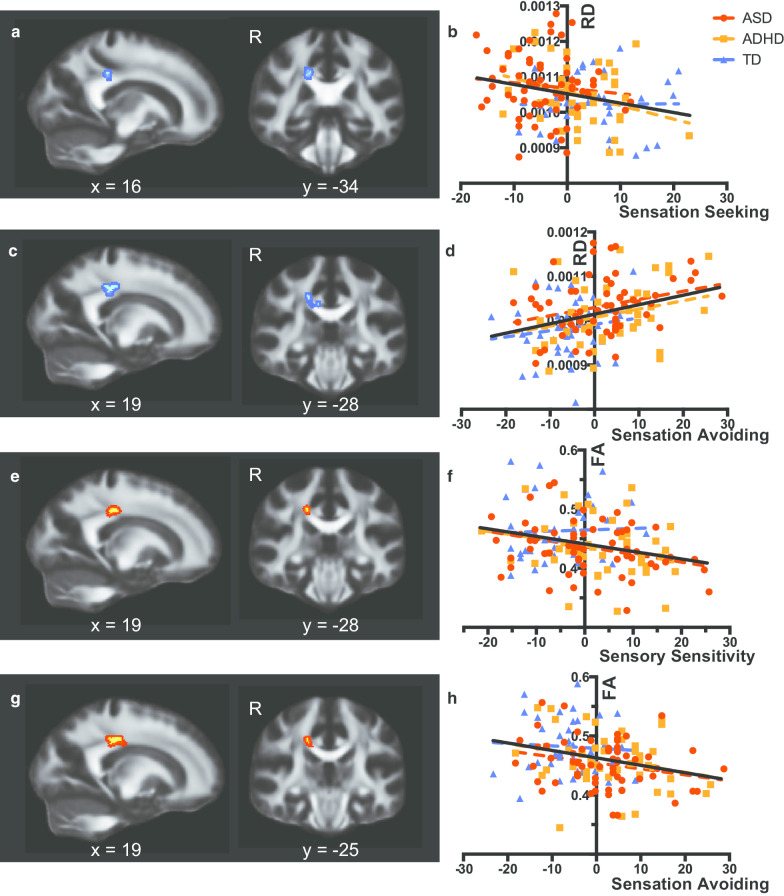
Significant voxels identified by dimensional analyses using subscale scores of the sensory profile. **a** Significant voxels identified by regression of radial diffusivity (RD) on the Sensation Seeking score. For the sake of visualization, the voxel clusters were thickened using the tbss_fill script implemented in FSL. **b** Scatterplots and regression lines showing relationships between the demeaned sensation seeking score and RD values extracted from voxels shown in **a** (*F*(1, 142) = 8.63, *f*^2^ = 0.061, *P* = 0.0039). Colored dotted lines indicate regression lines for the data of autism spectrum disorder (red), attention-deficit/hyperactivity disorder (orange), and typically developed participants (blue), whereas the black lines indicate regression lines for the combined data of the three groups. **c** Significant voxels identified by regression of RD on the Sensation Avoiding score. **d** Scatterplots and regression lines showing relationships between the demeaned Sensation Avoiding score and RD values. RD values were extracted from significant voxels in **c** (*F*(1, 141) = 15.17, *f*^2^ = 0.108, *P* < 0.001). **e** Significant voxels identified by regression of FA on the Sensory Sensitivity score. **f** Scatterplots and regression lines showing relationships between the demeaned Sensation Sensitivity score and FA values extracted from voxels shown in **e** (*F*(1, 142) = 10.70, *f*^2^ = 0.075, *P* = 0.001). **g** Significant voxels identified by regression of FA on the Sensation Avoiding score. **h** Scatterplots and regression lines showing relationships between the demeaned Sensation Avoiding score and FA values extracted from voxels shown in **G** (*F*(1, 141) = 11.75, *f*^2^ = 0.083, *P* < 0.001)

The dimensional analyses revealed that RD values in the posterior body of the corpus callosum were negatively associated with Sensation Seeking (Table 3 and Fig. 2a, b) and positively associated with Sensation Avoiding (Table 3 and Fig. 2c, d). We extracted mean RD values from identified voxels and confirmed that slopes in relationships of the AASP subscale with the DTI parameter were comparable among the three groups (*F*(2, 138) = 1.185, *η*^2^ = 0.0177, *P* = 0.31 for Fig. 2b; *F*(2, 137) = 0.036, *η*^2^ = 0.0088, *P* = 0.96 for Fig. 2d). The analysis with FA showed that the DTI parameter in the posterior body of the corpus callosum was negatively correlated with Sensory Sensitivity (Fig. 2e, f) and Sensation Avoiding (Table 3 and Fig. 2g, h). Slopes of the AASP subscale—FA relationships were comparable among the three groups (*F*(2, 138) = 0.90, *η*^2^ = 0.0301, *P* = 0.41 for Fig. 2f; *F*(2, 138) = 0.22, *η*^2^ = 0.0123, *P* = 0.80 for Fig. 2g).

**Table 3:** Significant clusters identified in dimensional or interaction models

White matter tract		MNI coordinate			Cluster size
*Dimensional analysis*					
RD and sensation seeking					
R. Posterior body of corpus callosum		16	− 34	35	17
RD and sensation avoiding					
R. Posterior body of corpus callosum		19	− 28	37	82
R. Posterior body of corpus callosum		16	− 32	32	34
FA and sensory sensitivity					
R. Posterior body of corpus callosum		19	− 28	37	30
FA and sensation avoiding					
R. Posterior body of corpus callosum		19	− 25	36	71
*Interaction analysis*					
FA and sensory sensitivity (ASD + ADHD vs. TD)					
R. Midbody of corpus callosum		11	− 2	30	73
RD and sensory sensitivity (ASD vs. ADHD)					
R. Posterior body of corpus callosum		16	− 35	37	13

### DTI interaction analyses

**Fig. 3:**
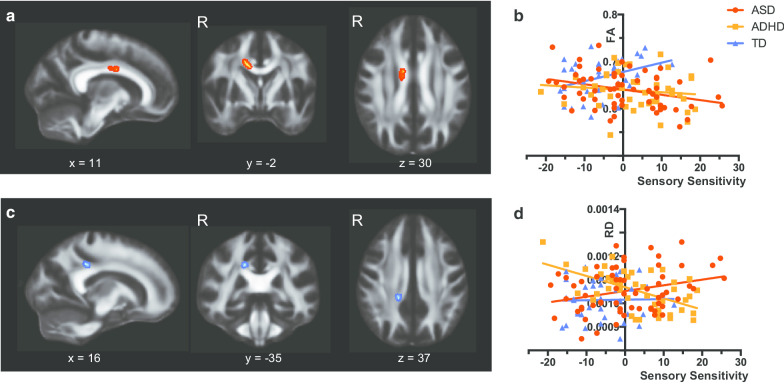
Significant voxels identified by interaction analyses of diagnosis status and subscale scores of sensory profile. **a** Significant voxels identified by the contrast between developmental disorders [autism spectrum disorder (ASD) and attention-deficit/hyperactivity disorder (ADHD)] and typically developed participants for the regression of fractional anisotropy (FA) on the Sensory Sensitivity score. For the sake of visualization, the voxel clusters were thickened using the tbss_fill script implemented in FSL. **b** Scatterplots and regression lines showing relationships between the demeaned Sensory Sensitivity score and FA values extracted from voxels in **a**. **c** Significant voxels identified by the contrast between ASD and ADHD for the regression of radial diffusivity (RD) on the Sensory Sensitivity score. **d** Scatterplots and regression lines showing relationships between the demeaned Sensory Sensitivity score and RD values extracted from voxels in **c**

Significant interaction in the Sensory Sensitivity–FA slope was observed between TD and DD groups. People with ASD had negative correlation between Sensory Sensitivity score and FA in the midbody of the corpus callosum, while TD people showed positive correlation (Table 3 and Fig. 3a, b). An *F* test confirmed significant between-group differences in the slopes of the FA-Sensory Sensitivity relationship (*F*(2, 138) = 5.57, *η*^2^ = 0.0746, *P* = 0.005). On the other hand, the analysis with RD showed significant interaction in Sensory Sensitivity score and RD value between ASD and ADHD groups (Table 3 and Fig. 3c, d). Individuals with ASD showed a positive correlation between RD value and Sensory Sensitivity scores, while individuals with ADHD had a negative correlation. We observed significant between-group differences in the slopes of the RD-Sensation Sensitivity relationship (*F*(2, 138) = 9.61, *η*^2^ = 0.159, *P* = 0.0001). The cluster was located in the right posterior corpus callosum. Other subscales of the AASP did not show any significant results in any DTI parameter.

### Supplementary analyses with a narrow age range with only males

#### Categorical analyses

Supplementary categorical analyses with only male individuals whose age was equal to or less than 40 years old (see Additional file 1: Table S2 for the demographic and clinical data of the three groups) showed the similar pattern to the primary categorical analyses (Additional file 1: Fig. S1). FA analysis showed a significant effect of diagnosis. Post hoc tests showed that the mean FA values in individuals with ASD and in individuals with ADHD were significantly lower than those in TD participants. In terms of RD, the analysis showed the main effect of diagnosis. Post hoc analyses showed lower RD values in TD participants compared with individuals with ASD and those with ADHD.

### DTI dimensional analyses

As shown in Additional file 1: Fig. S2, the pattern of the results remained substantially similar after narrowing the age range. More specifically, RD values in the posterior body of the corpus callosum were negatively associated with Sensation Seeking (Additional file 1: Fig. S2A) and positively associated with Sensation Avoiding (Additional file 1: Fig. S2B). Furthermore, in line with the primary analyses, FA values from the posterior corpus callosum were positively associated with Sensory Sensitivity and Sensation Avoiding (Additional file 1: Fig. S2C and S2D). Of note, in line with the analyses using a full age range, slopes of individuals with ASD and of those with ADHD were comparable in all the supplementary analyses (RD-Sensation Seeking: *F*(2, 108) = 1.08, *η*^2^ = 0.031, *P* = 0.34; RD-Sensation Avoiding: *F*(2, 108) = 0.417, *η*^2^ = 0.020, *P* = 0.66; FA-Sensory Sensitivity: *F*(2, 108) = 0.84, *η*^2^ = 0.033, *P* = 0.44; FA-Sensation Avoiding: *F*(2, 108) = 0.83, *η*^2^ = 0.032, *P* = 0.44).

### DTI interaction analyses

In the supplementary interaction analyses, the pattern of the results remained the same as in the primary analyses. In line with the primary analysis, the slope of FA-Sensory Sensitivity was positive in the TD controls, while it was negative in both the ASD and ADHD groups (Additional file 1: Fig. S3A). Group differences in the slopes of the FA-Sensory Sensitivity relationship were statistically significant (*F*(2, 108) = 5.44, *η*^2^ = 0.093, *P* = 0.006). In terms of the interaction between ASD and ADHD groups, the supplementary analysis showed a similar pattern as the primary analysis. RD values from the posterior corpus callosum area were positively correlated with Sensory Sensitivity in individuals with ASD, but negatively associated in individuals with ADHD (Additional file 1: Fig. S3B). We confirmed significant group differences in the slopes of the RD-Sensory Sensitivity relationship (*F*(2, 108) = 5.01, *η*^2^ = 0.103, *P* = 0.008).

## Discussion

The current TBSS study enrolled a relatively large number of participants: 218 adults (ASD: *n* = 105, ADHD: *n* = 55, TD: *n* = 58). In the conventional approach of categorical group comparisons, we used *F* tests and identified the main effects of diagnosis on FA and RD values in regions around the body and the splenium of the corpus callosum. Post hoc tests revealed that, compared with the TD group, FA values were lower in individuals with ASD and with ADHD, while they were not significantly different from each other. In terms of RD, individuals with ASD and with ADHD had higher values compared with the TD group, again, in the posterior part of the corpus callosum. The dimensional analysis, using the scores of sensory profiles, demonstrated an area in the isthmus of the corpus callosum where the three groups showed comparable relationships between the DTI parameters and sensory problems. In contrast, the interaction analyses showed significant results in a portion of the corpus callosum; the DD groups had a negative association between FA and Sensory Sensitivity, while the TD group showed a positive correlation. The interaction analysis with RD showed that individuals with ASD and those with ADHD had different associations between white matter organization and Sensory Sensitivity. These findings demonstrated both similarities and distinctions in DTI parameters and in relationships with sensory symptoms between the two developmental disorders of ASD and ADHD.

### Comparisons with previous transdiagnostic DTI studies of ASD and ADHD

Previously, two studies directly contrasted three groups using TBSS [14, 16]. Strikingly, Ameis et al. [14] identified the effect of diagnosis anatomically close to our current results. In addition, another study also reported anatomical overlap of the effect of diagnosis with the current study [14, 16]. Intriguingly, the current study and these two previous studies revealed the effect of diagnosis on DTI parameters in the right portion of the corpus callosum [16]. One possible explanation for this laterality is handedness. Another explanation is that the laterality of the brain itself may constitute the pathophysiology of ASD and ADHD [45, 46]. Indeed, atypical rightward cerebral asymmetry was associated with social reciprocity in ASD [45]. Although individuals with ASD are more likely to be non-right-handed [47], there is a paucity of ASD neuroimaging studies focusing on individuals who are non-right-handed. Future studies should focus on these individuals and disentangle the relationship between asymmetry and handedness.

Despite such an overlap of anatomical location, those two prior TBSS studies reported different results in ADHD [14, 16]. More specifically, Aoki et al. demonstrated that individuals with ADHD and TD were not statistically significantly different from each other [16]. On the other hand, Ameis et al. [14] reported lower FA values in individuals with ADHD, as compared with TD controls. Such inconsistency in FA findings in individuals with ADHD may be associated with age [13]. Indeed, DTI values are influenced by age [48]. More specifically, the FA value of the corpus callosum presents an inverted U-shaped curve across age in TD, peaking in young adulthood between 21 and 29 years of age (reviewed in [49]). In individuals with ADHD, a negative association between age and lower-than-typical FA value in the CC was reported [46]. Given that Aoki et al. recruited children aged between 6 and 13 [16], the current study enrolled adults aged between 20 and 55, and the age of the participants in the Ameis et al. [14] study is between the age range of these two studies, the inconsistent FA findings can be explained by the association between age and lower-than-typical FA findings in individuals with ADHD. Future research that fully depicts the developmental trajectory of DTI parameters in each brain region is expected.

### Sensory problems in developmental disorders

The present study participants with ADHD exhibited severe sensory symptoms to a degree comparable to, or even higher than, individuals with ASD in the three subscales (Low Registration, Sensory Sensitivity, and Sensation Avoiding). These results were not consistent with our expectations. However, they were fully consistent with the results from a study contrasting the scores of a similar measure called Sensory Profile [50] across people with ASD, ADHD, or TD, except for the scores for Sensation Seeking by Little and colleagues [51]. While the current study showed a significant difference in Sensation Seeking between individuals with ASD and with ADHD, Little et al. found no significant difference between them. Demographic differences in the participants could be one possible reason for this inconsistency. Little et al. recruited children and adolescents, while the current study recruited adults and middle-aged individuals. Medication status may also impact results. It should be noted that there is no established pharmacological treatment for sensory symptoms in individuals with ASD or those with ADHD [52]. However, the medication used to alleviate psychological symptoms, such as anxiety, would influence sensory symptoms. Our speculation is associated with neurotransmitters, such as GABA, altered in individuals with developmental disorders [53, 54] and involved in sensory processing [19, 55]. Given that medication influences neurotransmitter levels, medication may also influence sensory symptoms. Future studies are expected to explore novel approaches to treat sensory symptoms in developmental disorders.

### Dimensional and interaction analyses using scores of sensory problems

The dimensional analysis identified the body of the corpus callosum as being associated with sensory symptoms. Given that the region involves fibers connecting sensory areas of both hemispheres [56], it is reasonable to say that the anatomical location of the current results may correlate with sensory symptoms. It was striking that the best fit line in the analyses for individuals with ASD, with ADHD, and TD participants was quasi-parallel. These findings suggest that these brain regions were related to sensory symptoms, regardless of the clinical diagnosis. Given the possibility that sensory symptoms contribute to the development of ASD symptoms [19], the similarity in the brain-sensory symptoms relationships suggests that the process of developing ASD traits is shared by all three groups. However, it should be noted that similarity was claimed on the basis of there being no significant differences in the slopes of regression of a DTI parameter on a sensory symptoms score, not on the basis of rejecting a null hypothesis that the three lines statistically differed from each other.

The analysis showed significant interaction between Sensory Sensitivity and RD values in the posterior portion of the corpus callosum between individuals with ASD and those with ADHD. Among the four subscales, Sensory Sensitivity was the only subscale that showed such interaction. Four domains of AASP differed in interacting principles of neurological thresholds and behavioral responses [57]. Sensory Sensitivity was a combination of low neurological threshold and passive behavioral responses. Given that higher Sensory Sensitivity is atypical and that higher RD values are pathological, the pathological relationship in the ASD group was plausible. On the other hand, the ADHD group exhibited a negative association between RD in the corpus callosum and Sensory Sensitivity. Although the cause of the unexpected correlation in individuals with ADHD could not be addressed in the current study, the posterior portion of the corpus callosum is one of the hubs in the pathophysiology of both ASD and ADHD [12, 58]. A future longitudinal study should address the causal relationship between the corpus callosum and sensory symptoms.

## Limitations

There are some limitations in the current study. First, the age range of the participants is wide (from 20 to 55 years). Given that few studies have enrolled individuals with ASD in their forties or fifties, the current data might help to address the paucity of data for middle-aged individuals with ASD. However, a wide age range increases the heterogeneity of participants. As we included age as a covariate of nuisance and there was no significant difference in age between groups, the impact of this wide age range was minimized. Furthermore, we conducted supplementary analyses narrowing the age range, which showed a consistent pattern with the results of the primary analyses. Nevertheless, the results should be interpreted with caution, as the age range of the current study is wide. It should be noted that because of the nature of cross-sectional study, it was not possible to assume causal relationships between white matter and sensory symptoms. Future studies covering a wide age range should obtain longitudinal neuroimaging data to evaluate causal relationships between the corpus callosum and sensory symptoms. Besides age, there are some other factors that contribute to the heterogeneity of developmental disorders, such as functionality, comorbidity, and medication. Although we did not include people whose IQ was 75 or below, the inclusion criteria may not ensure biological homogeneity. In terms of comorbidity, we gave a priority to people with minimal comorbidities at recruitment, which is one of the strengths of the current research conducted at a research/clinical center specialized with the developmental disorders. However, formal and research-level diagnostic procedures were not conducted for other mental illnesses because of time and financial constraints. Recruitment of medication naïve participants was ideal to focus on the pathophysiology, which was not practically possible. These factors of origin of heterogeneity are also expected to be addressed in the future study. Second, we included both sexes. As we assumed that sex has an impact on the results [59], we included sex as a covariate of nuisance. Reflecting the known male predominant prevalence [60], the majority of our participants was male. Again, to increase biological homogeneity, we conducted supplementary analyses focusing on male participants aged between 20 and 40 years and found that the results were consistent with those of the primary analyses. However, the sample size was not large enough to repeat the analysis by including only female subjects. Future studies with larger sample sizes that include only females are needed to test the replicability of our findings. Thirdly, despite the fact that all the clinical population was recruited from the authors’ outpatient clinic, we did not obtain some important pieces of information on the participants, such as the employment status or living status at the time of the MRI scan. Although the employment status or living status often changes over time, factors of lifestyles potentially affect one's functionality and brain. Therefore, future studies need to more closely account for relationships of social and life factors with brain in developmental disorders. Finally, we had been meticulous to evaluate ASD traits in individuals with ADHD and vice versa. However, it is still possible that some individuals with ASD, who did not undergo full psychological evaluation, may have traits, symptoms, or even diagnosis of ADHD. This possibility does not affect the results of the dimensional analyses, but could impact the results of the interaction analyses. Ideally, all the participants, regardless of diagnoses, should have undergone evaluation of ASD and ADHD symptoms and diagnosis. However, because of financial restrictions and time constraints, it was not possible to conduct such a thorough evaluation. Future studies should address these limitations and should evaluate all participants, regardless of their diagnosis.

## Conclusions

The current DTI study enrolled adults whose primary diagnosis was ASD or ADHD and compared them to TD people to investigate similarities and differences in white matter organization. We investigated brain–behavior relationships from the perspective of sensory symptoms. This study showed that, in some brain regions, the three groups showed similar relationships of DTI parameters to sensory symptoms, while in other brain regions the groups showed different relationships between the DTI and sensory symptoms. The current study provided insight into similarities and distinctions in the process of development of clinical ASD symptoms and subclinical traits across ASD, ADHD, and TD.

## Supplementary information

**Additional file 1:** **Table S1.** Medications. **Table S2.** Descriptive statistics of the male subsample under 40 years of age. **Figure S1.** Post-hoc regions of interest analyses using the male-only subsample under 40 years of age. **Figure S2.** Post-hoc dimensional analyses using the male-only subsample under 40 years of age. **Figure S3.** Post-hoc regions of interest analyses showing significant interaction in the male-only subsample under 40 years of age.

## Data Availability

The datasets used and/or analyzed during the current study are available from the corresponding author on reasonable request.
