# Supplementary material for: White matter alterations in autism spectrum disorder and attention-deficit/hyperactivity disorder in relation to sensory profile
Source: Mol Autism. 2020 Oct 19;11:77. doi: 10.1186/s13229-020-00379-6 (PMC7570037; doi:10.1186/s13229-020-00379-6)
Supplement: Supplementary file 1 — Additional file 1: Table S1. Medications. Table S2. Descriptive statistics of the male subsample under 40 years of age. Figure S1. Post-hoc regions of interest analyses using the male-only subsample under 40 years of age. Figure S2. Post-hoc dimensional analyses using the male-only subsample under 40 years of age. Figure S3. Post-hoc regions of interest analyses showing significant interaction in the male-only subsample under 40 years of age. [file 13229_2020_379_MOESM1_ESM.docx]

Additional file 1

Table S1. Medications

| Medication | ASD n (%) | ADHD n (%) |
| --- | --- | --- |
| Antipsychotics | 13 (12%) | 2 (4%) |
| Atomoxetine | 5 (5%) | 13 (24%) |
| Benzodiazepine or Z-drugs | 19 (18%) | 5 (9%) |
| SNRI | 8 (8%) | 2 (4%) |
| SSRI | 8 (8%) | 4 (7%) |
| Stimulant | 4 (4%) | 30 (55%) |
| Tetracyclic antidepressant | 2 (2%) | 0 (0%) |
| Tricyclic antidepressant | 1 (1%) | 0 (0%) |
| Note: SNRI: ADHD: Attention-Deficit/Hyperactivity Disorder, ASD: Autism Spectrum Disorder, Serotonin & Norepinephrine Reuptake Inhibitors, SSRI: Selective Serotonin Reuptake Inhibitors | | |
|  |  |  |
|  |  |  |
|  |  |  |

Table S2. Descriptive statistics of the male subsample under 40 years of age

| Variable | ASD  (n=78) | ADHD  (n=36) | TD  (n=47) | *F or χ*^2^ statistic | Post-hoc test |
| --- | --- | --- | --- | --- | --- |
| Age, mean, SD, y | 28.8 (5.2) | 28.1 (5.3) | 28.0 (4.7) | *F*(2,158) = 0.45,  η^2^ = 0.006, *P* = 0.64 | NA |
| Handedness | 81.3 (48.8) | 70.6  (60.1) | 88.4  (38.8) | *F*(2, 158) = 1.35,  η^2^ = 0.017, *P* = 0.26 | NA |
| Head motion | 1.06 (0.102) | 1.05 (0.096) | 1.05 (0.100) | *F*(2, 158) = 0.19,  η^2^ = 0.002, *P* = 0.83 | NA |
| IQ | | | | | |
| Full | 107.7 (14.2) | 107.0 (12.5) | 108.7  (7.7) | *F*(2,152) = 0.19,  η^2^ = 0.002, *P* = 0.83 | NA |
| Verbal | 112.0  (14.2) | 109.6  (14.3) |  | *F*(1,106) = 0.61,  η^2^ = 0.006, *P* = 0.44 | NA |
| Performance | 100.2  (16.3) | 102.0  (13.3) |  | *F*(1,106) = 0.31,  η^2^ = 0.003, *P* = 0.58 | NA |
| AQ | 34.1  (5.9) | 29.5  (9.4) | 16.7  (5.9) | *F*(2,143)=101.6,  η^2^ = 0.611, *P* < 0.001 | ASD>ADHD>TD |
| CAARS | | | | | |
| Inattentive symptoms | 62.7  (13.7) | 75.5  (11.0) | 49.3  (8.5) | *F*(2,112) = 26.6,  η^2^ = 0.322, *P* < 0.001 | ADHD>ASD>TD |
| Hyperactive Impulsive Symptoms | 57.1  (12.9) | 65.8  (15.0) | 50.4  (9.7) | *F*(2,112) = 8.59,  η^2^ = 0.133 , *P* < 0.001 | ADHD>ASD=TD |
| ADHD Symptoms Total | 61.3  (13.4) | 73.0  (11.7) | 49.8  (8.8) | *F*(2,112) = 20.9,  η^2^ = 0.272, *P* < 0.001 | ADHD>ASD>TD |
| AASP | | | | | |
| Low Registration | 36.6  (8.1) | 39.0  (7.5) | 28.1  (6.7) | *F*(2,111) = 19.1,  η^2^ = 0.256, *P* < 0.001 | ADHD=ASD>TD |
| Sensation Seeking | 31.1  (5.3) | 36.5  (7.0) | 40.9  (7.8) | *F*(2,111) = 23.4,  η^2^ = 0.297, *P* < 0.001 | TD>ADHD>ASD |
| Sensory Sensitivity | 38.8  (9.9) | 41.3  (8.4) | 32.8  (7.7) | *F*(2,111) = 7.89,  η^2^ = 0.125, *P* < 0.001 | ADHD=ASD>TD |
| Sensation Avoiding | 39.6  (9.4) | 42.0  (10.2) | 32.2  (6.9) | *F*(2,111) = 10.8,  η^2^ = 0.162, *P* < 0.001 | ADHD=ASD>TD |

Figure S1


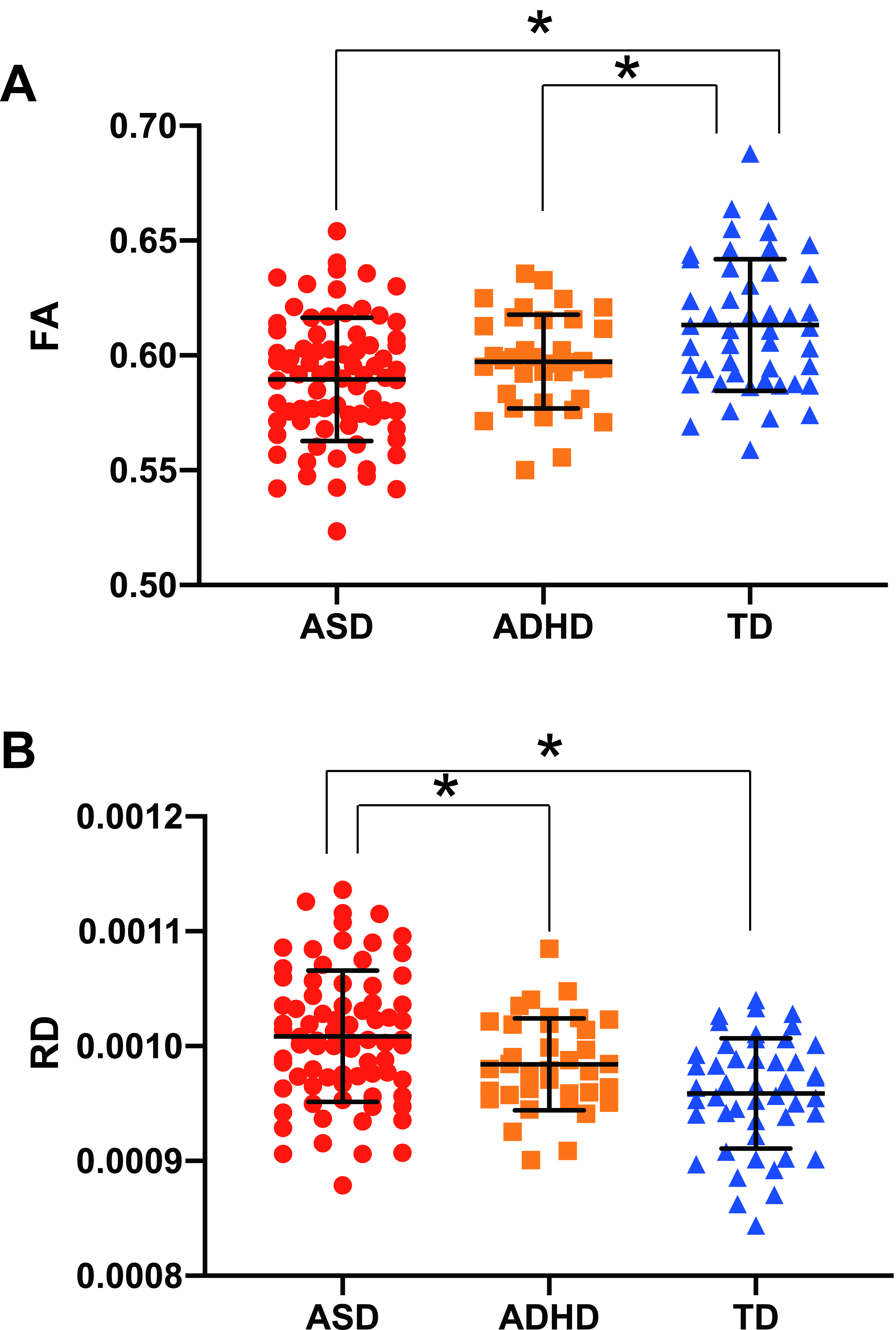


Figure S1. Post-hoc regions of interest analyses showing significant alterations of FA and RD values in developmental disorders using the male-only subsample under 40 years of age. (A) Plots of mean FA values extracted from significant voxels shown in Figure 1A in the main text. We confirmed a significant main effect of diagnosis (*F*(2, 158) = 11.99, *η*^2^ = 0.132, *P* < 0.001). (B) Plots of mean RD values extracted from significant voxels shown in Figure 1C in the main text. We confirmed a significant main effect of diagnosis (*F*(2, 158) = 14.03, *η*^2^ = 0.151, *P* < 0.001). The asterisk (*) indicates a significant difference between groups shown by Tukey's multiple comparison test (*P* < 0.05). Although the difference in RD between ADHD and TD did not reached the threshold, the group-difference remained marginally significant (*P* = 0.069).

Figure S2


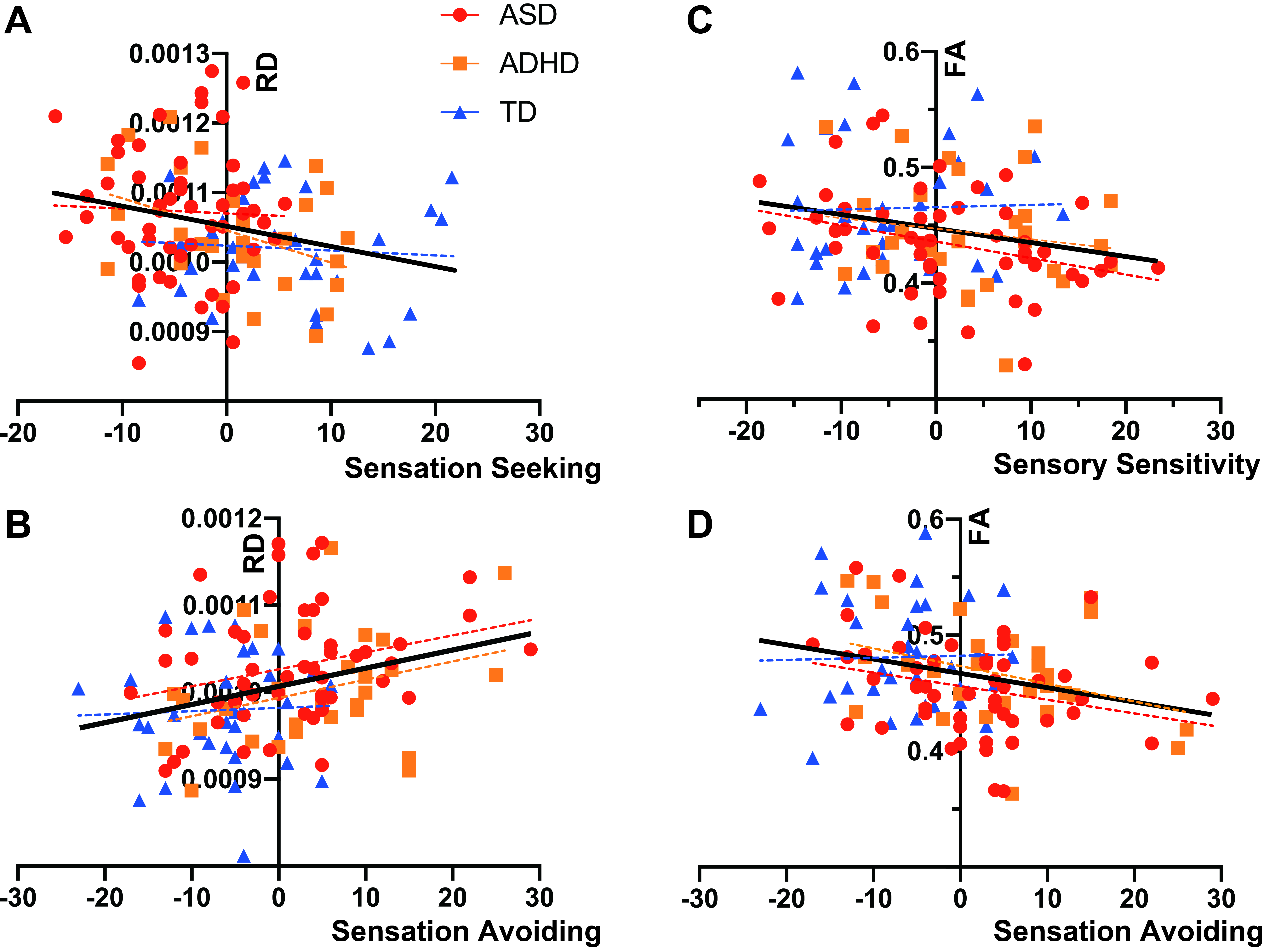


Figure S2. Post-hoc dimensional analyses using subscale scores of the sensory profile in the male-only subsample under 40 years of age. (A) Scatterplots and regression lines showing relationships between the demeaned Sensation Seeking score and radial diffusivity (RD) values extracted from voxels shown in Figure 2A in the main text (*F*(1, 112) = 7.73, *f*^2^ = 0.069, *P* = 0.0064). Colored dotted lines indicate regression lines for the data of autism spectrum disorder (red), attention-deficit/hyperactivity disorder (orange), and typically developed participants (blue), whereas the black lines indicate regression lines for the combined data of the three groups. (B) Scatterplots and regression lines showing relationships between the demeaned Sensation Avoiding score and RD values. RD values were extracted from significant voxels in Figure 2C (*F*(1, 112) = 10.41, *f*^2^ = 0.093, *P* = 0.0016). (C) Scatterplots and regression lines showing relationships between the demeaned Sensation Sensitivity score and FA values extracted from voxels shown in Figure 2E (*F*(1, 112) = 6.44, *f*^2^ = 0.058, *P* = 0.0125). (D) Scatterplots and regression lines showing relationships between the demeaned Sensation Avoiding score and FA values extracted from voxels shown in Figure 2G (*F*(1, 112) = 8.72, *f*^2^ = 0.078, *P* = 0.0038).

Figure S3


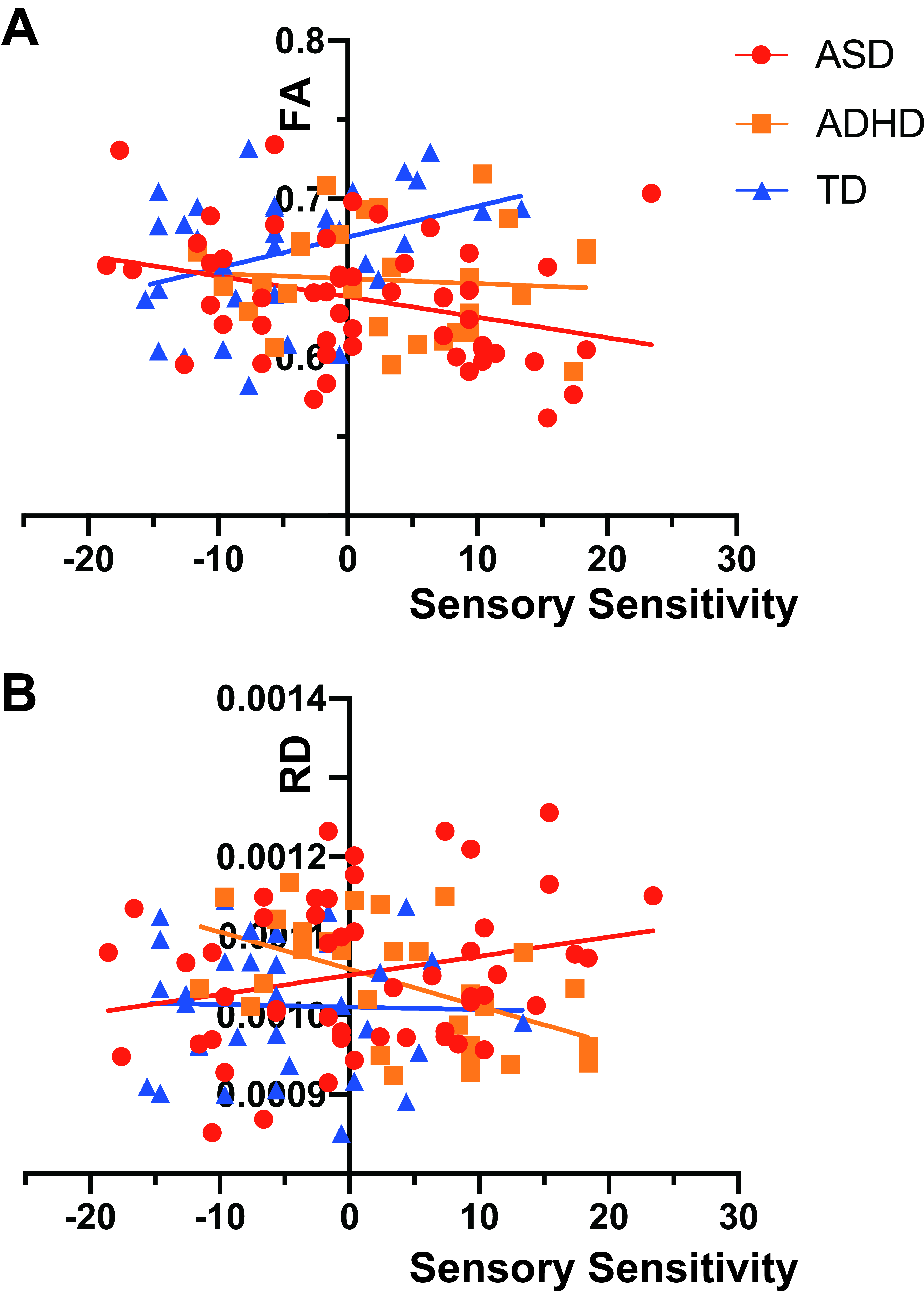


Figure S3. Post-hoc regions of interest analyses showing significant interaction between diagnosis status and subscale scores of sensory profile. (A) Scatterplots and regression lines showing relationships between the demeaned Sensory Sensitivity score and fractional anisotropy (FA) values extracted from voxels in Figure 3A in the main text. (B) Scatterplots and regression lines showing relationships between the demeaned Sensory Sensitivity score and RD values extracted from voxels in Figure 3C in the main text.
